# Downregulation of the Long Non-Coding RNA *KLRK1-AS1* Disturbs Endothelial Barrier Integrity and Promotes Angiogenic Sprouting

**DOI:** 10.3390/life16020279

**Published:** 2026-02-05

**Authors:** Elisa Weiss, Azra Kulovic-Sissawo, Anke S. van Bergen, Veerle Kremer, Mariana S. Diniz, Carolina Tocantins, Susana P. Pereira, Reinier A. Boon, Ursula Hiden

**Affiliations:** 1 Research Unit Early Life Determinants (ELiD), Medical University of Graz, Auenbruggerplatz 14, 8036 Graz, Austria; 2 Department of Physiology, Amsterdam University Medical Centers, De Boelelaan 1108, 1081HZ Amsterdam, The Netherlands; a.vanbergen@amsterdamumc.nl (A.S.v.B.);; 3 Amsterdam Cardiovascular Sciences, Microcirculation, Meibergdreef 9, 1105AZ Amsterdam, The Netherlands; 4 CNC-UC Center for Neuroscience and Cell Biology, University of Coimbra, Rua Larga, 3004-504 Coimbra, Portugal; 5 CIBB-Centre for Innovative Biomedicine and Biotechnology, University of Coimbra, Rua Larga, 3004-504 Coimbra, Portugal; 6 Institute for Interdisciplinary Research (IIIUC), Doctoral Programme in Experimental Biology and Biomedicine (PDBEB), University of Coimbra, Rua Larga, 3004-504 Coimbra, Portugal; 7 UCIBIO—Applied Molecular Biosciences Unit, Department of Chemistry, NOVA School of Science and Technology (NOVA FCT), NOVA University of Lisbon, 2829-516 Caparica, Portugal; sps.pereira@fct.unl.pt; 8 Associate Laboratory i4HB, Institute for Health and Bioeconomy, NOVA School of Science and Technology (NOVA FCT), NOVA University of Lisbon, 2829-516 Caparica, Portugal; 9 Institute for Cardiovascular Regeneration, Centre for Molecular Medicine, Goethe University Frankfurt am Main, Theodor-Stern-Kai 7, 60590 Frankfurt am Main, Germany; 10 German Centre for Cardiovascular Research DZHK, Partner Site Frankfurt Rhein/Main, 60590 Frankfurt am Main, Germany

**Keywords:** long non-coding RNA (lncRNA), endothelial colony-forming cell (ECFC), proliferation, barrier integrity, angiogenesis, *KLRK1-AS1*, cardiovascular health

## Abstract

Endothelial integrity is essential for cardiovascular health, and circulating endothelial progenitor cells, particularly endothelial colony-forming cells (ECFCs), are key contributors to vascular repair and maintenance. Long non-coding RNAs (lncRNAs) have emerged as novel epigenetic regulators of endothelial physiology and pathology. Building on our previous work identifying the lncRNA *KLRK1-AS1* as a positive modulator of ECFC wound healing, we aimed to elucidate its role in endothelial biology. Cord blood-derived ECFCs were subjected to siRNA-mediated silencing of *KLRK1-AS1*, followed by blinded evaluations of monolayer morphology, barrier stability using ECIS impedance measurements, assessments of proliferation, and spheroid-based angiogenic activity. SiRNA-mediated silencing of *KLRK1-AS1* induced detectable alterations in ECFC monolayer morphology (*p* = 0.047), while proliferation remained unaffected. Notably, *KLRK1-AS1* knockdown significantly compromised endothelial barrier integrity, resulting in a 44% reduction in impedance after 48 h (*p* < 0.001), suggesting weakened intercellular contacts. In contrast, loss of *KLRK1-AS1* enhanced angiogenic behaviour, demonstrated by an increased number of sprouts (+62%, *p* = 0.031). Together, these findings indicate that *KLRK1-AS1* supports a quiescent, stable endothelial phenotype, with intact barrier function, while its depletion shifts ECFCs toward a more angiogenic, activated state. Our results identify *KLRK1-AS1* as a previously unrecognised regulator of endothelial function.

## 1. Introduction

The endothelium lines the entire vascular system and fulfils a wide range of essential physiological functions. Beyond acting as a simple physical lining, endothelial cells form a dynamic, semi-permeable barrier that regulates the exchange of fluids, solutes, and cells between the bloodstream and surrounding tissues. In addition, endothelial cells actively participate in immune responses, support angiogenesis, regulate vascular tone, and contribute to coagulation control. Under physiological conditions, endothelial cells maintain a quiescent state characterised by anti-inflammatory, vasodilatory, and anti-thrombotic properties [1,2]. This quiescent phenotype is critical for preserving vascular homeostasis and preventing inappropriate vascular responses. In contrast, exposure to stimuli such as inflammatory mediators or metabolic stress can induce endothelial activation. Activated endothelial cells display reduced barrier integrity, increased permeability, altered morphology, and enhanced pro-angiogenic behaviour—features that collectively define endothelial dysfunction [1,2]. Endothelial dysfunction is a key pathological process underlying the initiation and progression of cardiovascular diseases (CVDs), including atherosclerosis, coronary, carotid and peripheral artery disease, cardiorenal syndrome, and ischemic stroke [3,4,5]. Thus, understanding the molecular mechanisms that preserve endothelial quiescence or promote endothelial activation is central to cardiovascular research.

However, rather than representing binary states, endothelial quiescence and activation should be viewed as a dynamic and reversible continuum. Endothelial cells exhibit pronounced phenotypic plasticity, allowing them to rapidly adapt their functional properties in response to environmental cues such as inflammatory mediators, metabolic stress, and mechanical forces. Depending on the nature, strength, and duration of these stimuli, endothelial cells can transition between stable, barrier-forming phenotypes and more migratory, angiogenic states. This plasticity is essential for physiological processes such as vascular repair and tissue regeneration but may become maladaptive when chronically activated, thereby contributing to endothelial dysfunction. Understanding molecular regulators that govern this phenotypic balance is therefore critical for elucidating mechanisms underlying vascular homeostasis and disease.

Circulating endothelial progenitor cells (EPCs) play a central role in maintaining endothelial integrity and contribute to vascular repair and regeneration [6,7]. During neovascularisation and vascular repair, EPCs are recruited from the bloodstream [8]. Through these actions, EPCs exert protective effects against endothelial dysfunction and the progression of CVD [8]. Among EPC subtypes, endothelial colony-forming cells (ECFCs) represent a distinct population that is uniquely capable of differentiating into fully mature endothelial cells [9]. ECFCs are characterised by their ability to integrate into endothelial monolayers, their high proliferative potential, and their pronounced angiogenic capacity [10]. In addition to peripheral blood, ECFCs can be isolated from umbilical cord blood, where they occur in particularly high abundance and exhibit robust functional properties [11]. For these reasons, cord blood-derived ECFCs constitute a well-established and physiologically relevant model to study endothelial function and dysfunction.

Similar to endothelial cells of the vascular wall, ECFCs are adversely affected by cardiovascular risk factors (CVRFs), including weight gain, diabetes, and smoking, which impair their functional capacity [8]. Consequently, ECFCs are reduced in abundance and exhibit functional impairments in patients with CVD [8]. Umbilical cord blood-derived ECFCs are likewise sensitive to CVRF exposure during pregnancy and display disturbed functional properties in vitro [12]. Together, these characteristics make ECFCs a suitable and informative tool to investigate endothelial function and dysfunction.

Endothelial function and activation are tightly regulated by a complex network of molecular signals, including growth factors [13,14], hormones [15,16] and microRNAs (miRNAs) [17]. In recent years, long non-coding RNAs (lncRNAs) have emerged as additional regulators of endothelial homeostasis and dysfunction [18]. LncRNAs are defined as RNA transcripts longer than 200 nucleotides that do not encode conventional proteins. Based on their genomic origin and orientation, lncRNAs can be classified as intergenic, intronic, sense, or antisense transcripts [19]. Furthermore, lncRNAs may act in cis to regulate neighbouring genes or in trans to influence distant targets [19].

Mechanistically, lncRNAs exert their regulatory functions through diverse pathways. These include transcriptional regulation via modulation of transcription factor binding or chromatin structure, post-transcriptional effects on splicing or translation, and interactions with miRNAs or protein complexes involved in RNA-induced silencing [20]. Due to this mechanistic diversity, lncRNAs have been implicated in a broad range of diseases, including cancer [19], and are increasingly recognised as regulators of endothelial biology and dysfunction [20,21,22] and the associated CVD [18]. However, for many lncRNAs, their precise role in endothelial physiology remains incompletely understood.

We recently identified the lncRNA *KLRK1-AS1* as being downregulated in neonatal ECFCs when mothers experience excessive gestational weight gain [23]. Weight gain in general, even if modest, is considered a CVRF [24], and excessive gestational weight gain increases cardiovascular risk during [25] and after pregnancy [26]. Based on these observations, we proposed a link between elevated gestational weight gain, reduced *KLRK1-AS1* expression, and impaired endothelial function. Supporting this hypothesis, in vitro experiments demonstrated that reduced *KLRK1-AS1* levels diminish the wound-healing capacity of ECFC monolayers [23].

*KLRK1-AS1* (also known as LOC101928100, ENSG00000245648) overlaps with the *KLRK1* gene but is transcribed from the antisense strand on chromosome 12. Multiple isoforms have been described, ranging from 473 to 3388 base pairs in length. *KLRK1-AS1* is expressed in various human tissues, particularly but not exclusively including internal, secretory and reproductive organs (https://www.genecards.org, accessed on 15 January 2026). Dysregulated expression of *KLRK1-AS1* has been reported in several pathological contexts, including non-small cell lung cancer [27], lung squamous cell carcinoma [28] and neuroblastoma [29], and non-smoking chronic obstructive pulmonary disease (COPD), where it acts through interactions with other lncRNAs and miRNAs [30]. Moreover, *KLRK1-AS1* has been linked to neurological disorders through interactions with its host gene *KLRK1* [31]. Some *KLRK1-AS1* isoforms encode the peptide TP53LC04, a tumour protein P53 (TP53)-regulated micropeptide that suppresses cell proliferation [32]. These findings highlight the versatile nature of *KLRK1-AS1* and suggest that it may influence cellular function through multiple mechanisms. The genomic organisation of *KLRK1-AS1* isoforms within the *KLRK1* gene and the intron encoding TP53LC04 is shown in Supplementary Figure S1.

Building on our previous findings that implicate *KLRK1-AS1* in ECFC wound healing, the present study aims to explore its broader functional relevance in ECFC biology. Specifically, we investigated the impact of *KLRK1-AS1* on ECFC proliferation, barrier integrity, and angiogenic capacity to clarify its role in maintaining endothelial function and shaping endothelial phenotype.

## 2. Materials and Methods

### 2.1. Ethical Approval

This study was approved by the ethics committee of the Medical University of Graz, Austria (29-319 ex 16/17) in accordance with the Declaration of Helsinki. Written informed consent was obtained from all participants.

### 2.2. ECFC Isolation and Culture

Cord blood for the isolation of ECFCs used in this study was collected between 1 January 2018 and 1 January 2020 Samples were pseudonymized by research nurses, and only information regarding the absence of pathologies or medication intake during pregnancy, as well as the neonatal sex, was provided to the research laboratory along with the samples. Only samples from full-term, singleton and healthy pregnancies were included. No additional information was required. Venous umbilical cord blood was collected immediately after delivery to isolate ECFCs, as previously described [33]. Ten individual ECFC isolations were established, with six isolations from umbilical cord blood of male and four isolations from umbilical cord blood of female neonates. Cells from both sexes were used in all experiments. The number of cells isolations used from this cohort is indicated for every individual experiment and ranges between 6 and 7. After isolation and expansion, ECFCs were subjected to quality control by positive immunocytochemical staining for the endothelial cell markers CD31 and von Willebrand factor (VWF), and by negative staining for the fibroblast marker CD90 and smooth muscle actin. Representative stainings are shown in Supplementary Figure S2. Then, wells were cryopreserved in culture medium supplemented with 20% FCS (Thermo Fisher Scientific, Waltham, MA, USA) and 10% DMSO (SERVA, Heidelberg, Germany), and stored in liquid nitrogen until use. For experiments, ECFCs from passages 5–7 were cultured in Endothelial Cell Medium (ScienCell, Carlsbad, CA, USA) supplemented with Endothelial Cell Growth Supplement and 5% foetal bovine serum (FBS; ScienCell) at 37 °C, 21% O_2_, 5% CO_2_ in a humidified incubator. All cell preparations tested negative for mycoplasma contamination (MycoAlert™ mycoplasma detection kit; Lonza, Basel, Switzerland).

### 2.3. SiRNA Transfection

Transfection with specific siRNAs was performed as previously described [23]. In brief, ECFCs were transfected with 50 nM siRNA (Sigma-Aldrich, St. Louis, MO, USA; Sense: GAUCAGAGAAAGAAGCAUA, Antisense: UAUGCUUCUUUCUCUGAUC) using Lipofectamine RNAiMax Transfection Reagent (Invitrogen, Waltham, MA, USA) and OptiMEM Reduced Serum Medium supplemented with GlutaMAX (Gibco, Thermo Fisher Scientific). The siRNA binds to all currently known *KLRK1-AS1* isoforms. A non-targeting siRNA (MISSION siRNA Universal Negative Control #1, Sigma-Aldrich) served as an unspecific control. Then, 24 h after transfection, cells were seeded for subsequent experiments and RNA extraction to confirm knockdown efficiency. Supplementary Figure S1 illustrates the different *KLRK1-AS1* isoforms with the introns targeted by the antisense RNA highlighted.

### 2.4. RT-qPCR

Knockdown efficiency following siRNA transfection was assessed by RT-qPCR. Total RNA was extracted using the Direct-zol RNA miniprep kit (Zymo Research, Irvine, CA, USA) after cell lysis in TRIzol (Thermo Fisher Scientific). One µg of RNA was reverse-transcribed using the iScript cDNA synthesis kit (BioRad, Hercules, CA, USA) and RT-qPCR was subsequently performed with the iQ SYBR Green Supermix (BioRad) on a CFX384 or CFX96 Touch Real-Time PCR Detection System (BioRad). Oligo(dT) primers (5′–3′, Sigma-Aldrich) were used to amplify *KLRK1-AS1* (forward TGAAACGGATTCCCATGGCT, reverse TGCTTCTTTCTCTGATCTGTGTCT) with expression normalised to *RPLP0* (forward TCGACAATGGCAGCATCTAC, reverse ATCCGTCTCCACAGACAAGG). Gene expression was quantified using the 2^−ΔΔCt^ method. All samples were analysed in duplicates.

### 2.5. BrdU ELISA

Cell proliferation was assessed by measuring DNA synthesis using a colorimetric enzyme-linked immunosorbent assay (ELISA) based on 5′-bromo-2′-deoxyuridine (BrdU) incorporation (Roche, Basel, Switzerland), performed according to the manufacturer’s instructions. Briefly, 15,000 cells were seeded per well in 100 µL of culture medium in a 96-well plate. After 24 h, the assay was performed in six replicates by adding 10 µL of BrdU solution (100 µM) to each well and incubating for 3 h. Subsequently, the medium was removed and cells were fixed with 200 µL of FixDenat solution for 30 min. Thereafter, 100 µL of peroxidase-conjugated anti-BrdU antibody solution was added and incubated for 90 min in the dark. Following washing steps, 100 µL of substrate solution was added and incubated for another 15 min in the dark. Absorbance was measured in a SPECTROstar Nano absorbance reader (BMG Labtech, Ortenburg, Germany) at 370 nm with a reference wavelength of 492 nm. For analysis, slopes of optical density (OD) values, obtained from kinetic measurements recorded until reaching a plateau, were used.

### 2.6. Barrier Integrity and Proliferation Measurement via ECIS

Endothelial barrier integrity was assessed using the Electrical Cell–Substrate Impedance Sensing (ECIS) system (Applied Biophysics, Troy, NY, USA). ECIS allows continuous, non-invasive monitoring of endothelial monolayer formation and barrier stability by measuring the electrical impedance of cells grown on gold electrodes. For that purpose, 96-well arrays (96W10idf PET; Ibidi, Graefelfing, Germany) were pre-treated with 10 mM L-cysteine (Sigma-Aldrich) and coated with 1% gelatine (Merck, Darmstadt, Germany) to promote uniform cell attachment. ECFCs were seeded at a density of 40,000 cells per well, and impedance was recorded continuously for 48 h. Blank-corrected hourly resistance values at 4000 Hz were used to assess general barrier integrity. Using the ECIS software v1.2.210.0 PC, impedance data were modelled to extract the parameters Rb and Alpha, which provide mechanistic insight into barrier properties. The Rb parameter reflects resistance at cell–cell junctions and thus primarily represents the integrity of intercellular contacts, whereas the Alpha parameter is associated with cell–substrate interactions and provides information on cell–matrix adhesion and basolateral attachment. Blank-corrected hourly capacitance values at 64,000 Hz were used to assess proliferation-related changes in cell coverage, as higher frequencies are less sensitive to junctional resistance and more strongly influenced by cell number and electrode coverage. All measurements were performed in quadruplicates.

### 2.7. Spheroid Sprouting Assay

Angiogenic capacity was evaluated using the spheroid sprouting assay [34]. ECFCs (500 cells per well in 100 µL) were seeded into 96-well U-bottom plates (Costar, Corning, Corning, NY, USA) in culture medium supplemented with 0.6 g/L methylcellulose (Sigma Aldrich) and incubated for 24 h at 37 °C, 21% O_2_, 5% CO_2_ in a humidified incubator. Formed spheroids were embedded in a 1:1 mixture consisting of FBS (ScienCell) containing 2.4 g/L methylcellulose and a collagen solution composed of 3.77 g/L collagen I (Corning), 10% M199 medium (10×; Sigma Aldrich), 0.018 M HEPES and 0.02 M NaOH (pH 7.4). The mixture was distributed into two wells of a 24-well plate and allowed to polymerize at 37 °C, 21% O_2_, 5% CO_2_. After 30 min, 100 µL of culture medium was added on top of the gel, and plates were returned to the incubator. After 24 h, spheroids were fixed with 10% formaldehyde (diluted in PBS) and imaged using an Olympus IX50 (Tokyo, Japan) microscope. The number of sprouts and cumulative sprout length were selected as complementary quantitative readouts of angiogenic activity. While the number of sprouts reflects the initiation of angiogenic outgrowth and the ability of endothelial cells to form new protrusions, cumulative sprout length provides information on the extent and persistence of sprouting and thus overall angiogenic capacity. The number of sprouts and cumulative sprout length were quantified using ImageJ version 1.49v.

### 2.8. Data Analysis and Statistics

Statistical analyses were performed using JASP 0.95.4 software (University of Amsterdam, Amsterdam, The Netherlands), and figures were generated using GraphPad Prism (version 9). The sample size (*n*) refers to the number of individual ECFC donors transfected with *siKLRK1-AS1* and control siRNA, respectively. For all analyses, mean values of technical replicates derived from the same donor were used, ensuring that statistical inference was performed at the donor level rather than the technical replicate level. Given the paired experimental design (control vs. siRNA-treated ECFCs derived from the same donor) and the limited sample size, non-parametric statistical methods were chosen for group comparisons. Specifically, the Wilcoxon signed-rank test was used, as it does not rely on assumptions of normality and is well suited for paired data. For graphical presentation, data are shown as mean ± standard deviation (SD) with individual donor values displayed, as Shapiro–Wilk testing indicated approximate normal distribution. Time-resolved impedance data obtained from ECIS measurements were analysed using linear mixed-effects model (LMM) analysis. This approach was selected to appropriately account for repeated measurements over time and inter-donor variability. In the LMM, treatment, time, and their interaction (treatment × time) were included as fixed effects, while donor identity was specified as a random effect. This model structure allows separation of within-donor treatment effects from between-donor variability and provides robust inference for longitudinal data with unequal variances or missing values. A *p* value < 0.05 was considered statistically significant. ECFCs were isolated from umbilical cord blood of both male and female neonates. Although no sex-specific differences in knockdown effects were observed, the sample size in functional assays was insufficient to formally assess sex-related effects, and thus sex was not included as a fixed factor in the statistical models.

## 3. Results

### 3.1. KLRK1-AS1 Silencing Alters ECFC Morphology

**Figure 1:**
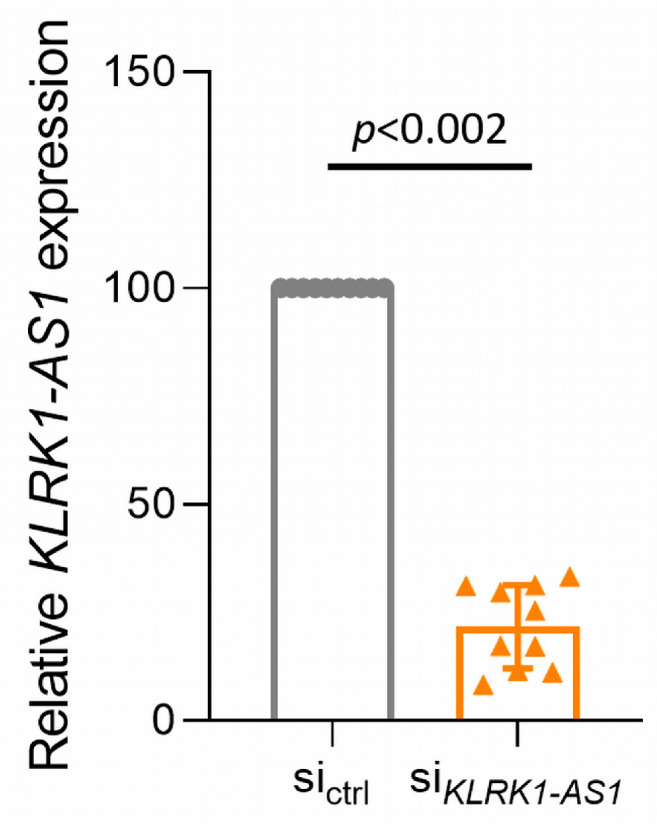
Silencing of ECFCs with siRNA for *KLRK1-AS1* reduces its expression. Statistics used Wilcoxon signed-rank test for raw data (ΔCt values) 48 h after transfection. Relative expression is shown as mean ± SD of *n* = 10 donors.

Efficient silencing of *KLRK1-AS1* was confirmed 48 h post-transfection by RT-qPCR, revealing a 78% reduction compared with non-specific control siRNA (*p* < 0.002). Relative expression levels (2^−ΔΔCt^) of *KLRK1-AS1* after silencing are shown in Figure 1; statistical analysis used the ΔCt values.

**Figure 2:**
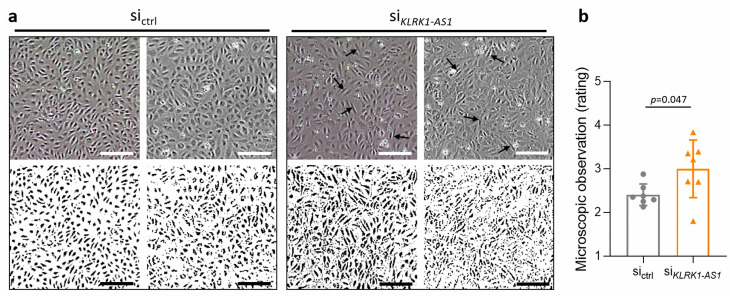
*KLRK1-AS1* silencing alters ECFC monolayer morphology. (**a**) Representative images demonstrating the elongated morphology and more irregular monolayer organisation in *KLRK1-AS1*-silenced ECFCs compared with control siRNA-treated cells. Elongated cells are indicated by arrows. Lower panel: images with maximum contrast enhancement. Scale bar = 200 µm. (**b**) Morphology scores obtained from blinded evaluation of ECFCs from seven donors transfected with *KLRK1-AS1* or control siRNA. Scores ranged from 1 (cobblestone morphology) to 5 (elongated shape). Data are presented as mean ± SD of *n* = 7 donors; statistical significance was assessed using the Wilcoxon signed-rank test.

Microscopic inspection revealed that *KLRK1-AS1* silencing induced notable morphological changes in ECFCs, characterised by a more elongated cell shape and an irregular monolayer pattern (Figure 2a, upper panel). To facilitate visualisation of these differences, maximum contrast enhancement was applied to the images (Figure 2a, lower panel). To quantitatively assess these observations, anonymised images from seven ECFC donors were independently evaluated by five blinded observers. Using a scoring system ranging from one (cobblestone morphology) to five (elongated phenotype), *KLRK1-AS1*-silenced ECFCs received significantly higher morphology scores compared with controls (*p* = 0.047; Figure 2b), demonstrating a more compact endothelial morphology in presence of *KLRK1-AS1*. The reference scale used for scoring is shown in Supplementary Figure S3.

### 3.2. KLRK1-AS1 Silencing Impairs ECFC Barrier Function

**Figure 3:**
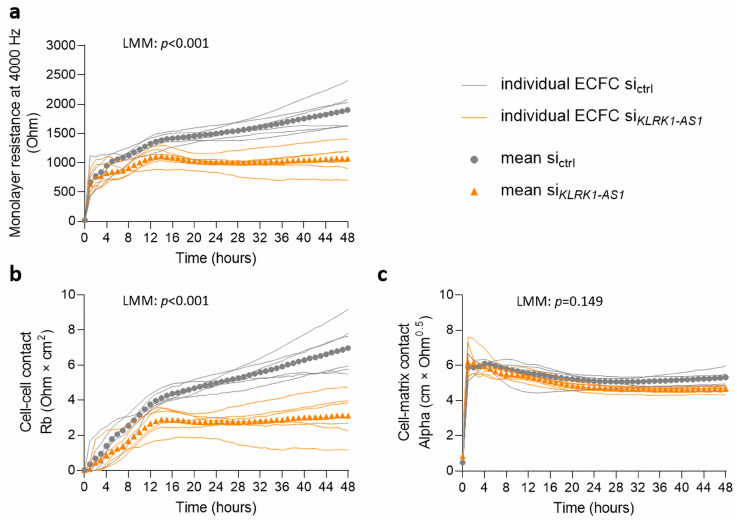
*KLRK1-AS1* silencing impairs barrier integrity in ECFCs. (**a**) Impedance measurements showing reduced monolayer barrier function after *KLRK1-AS1* silencing. (**b**) Decreased cell–cell contacts (Rb), with (**c**) unchanged cell–matrix adhesion (Alpha). Individual donor trajectories and descriptive means are shown for visualisation. Statistical inference was performed using linear mixed effects models (LMM) accounting for repeated measurements within donors. The *p*-value refers to a significant difference in the interaction between treatment (silencing) and time. *n* = 6 ECFC donors.

To assess whether *KLRK1-AS1* influences endothelial barrier properties, electrical impedance measurements were performed using the ECIS system. A stable impedance plateau, reflecting the formation of a confluent monolayer, was reached approximately 48 h after cell seeding. LMM analysis revealed a significant (*p* < 0.001) interaction between *KLRK1-AS1* silencing and time (with the model-estimate effect β = 8.23 and its SE = 0.74), indicating a time-dependent effect of *KLRK1-AS1* silencing on barrier resistance (Figure 3a). Further parameter modelling using ECIS software revealed that this reduction was primarily attributable to impaired cell–cell contacts, as indicated by a significant decrease in the intercellular resistance parameter Rb (LMM: *p* < 0.001, β = 0.038, SE = 0.003; Figure 3b). In contrast, cell–matrix interactions, represented by the Alpha parameter, remained unchanged (LMM: *p* = 0.149, β = 0.007, SE = 0.004; Figure 3c).

### 3.3. KLRK1-AS1 Silencing Does Not Affect ECFC Proliferation

**Figure 4:**
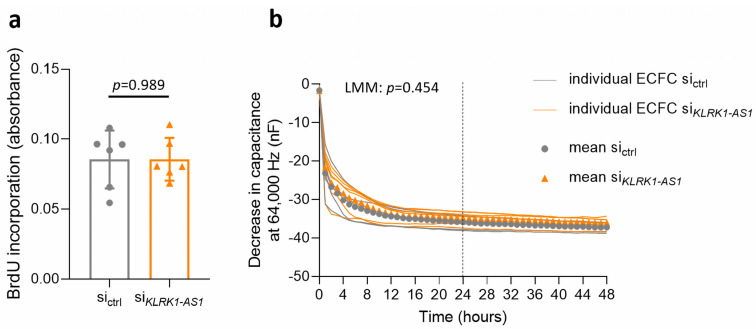
*KLRK1-AS1* silencing does not affect ECFC proliferation. (**a**) BrdU ELISA analysis showed no difference in BrdU incorporation between ECFCs transfected with *KLRK1-AS1*-specific siRNA and those transfected with control siRNA. Group comparison of BrdU incorporation was performed using Wilcoxon signed-rank test and data are presented as mean ± SD. (**b**) Capacitance measurement of the monolayer at 64,000 Hz as proxy for cell proliferation also did not reveal an effect of *KLRK1-AS1* silencing. Individual donor trajectories and descriptive means are shown for visualisation. Statistical inference of capacitance over time was performed using linear mixed-effects models (LMM) accounting for repeated measurements within donors. As the readout was proliferation, only the timecourse of the first 24 h, covering the logarithmic increase, was used for analysis, indicated by the dashed line. The *p*-value refers to the interaction between silencing and time. n = 6 ECFC donors.

To determine whether *KLRK1-AS1* influences proliferation of ECFCs, we quantified DNA synthesis using a BrdU ELISA. *KLRK1-AS1* silencing did not alter BrdU incorporation compared with control cells (*p* = 0.989; Figure 4a), indicating unchanged proliferative capacity. In addition, high-frequency ECIS measurements were analysed as an indirect proxy for proliferation. Although the capacitance under *KLRK1-AS1* silencing was slightly lower, LMM analysis did not indicate a statistically significant difference (*p* = 0.454; Figure 4b). Collectively, these results suggest that *KLRK1-AS1* does not substantially modulate ECFC proliferation under the conditions tested.

### 3.4. KLRK1-AS1 Silencing Promotes ECFC Angiogenic Sprouting

**Figure 5:**
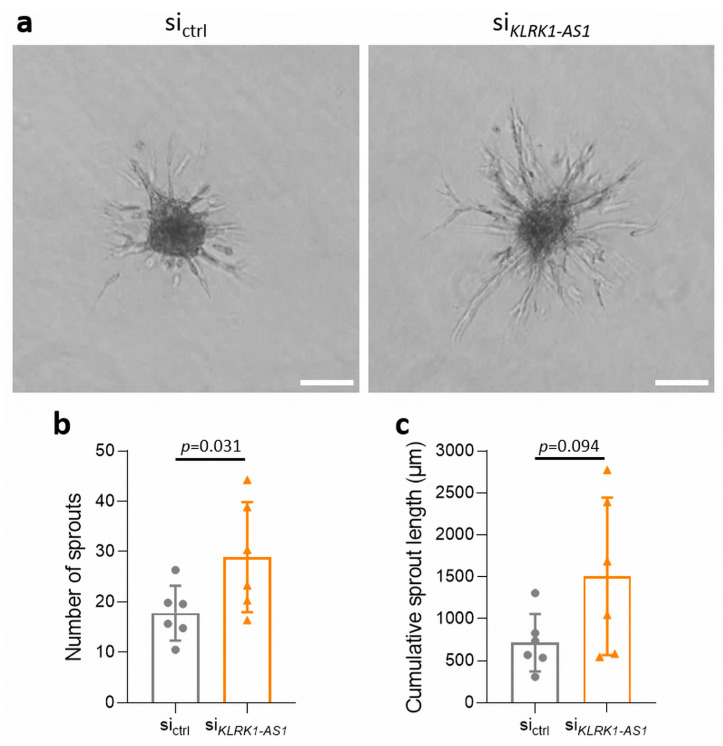
*KLRK1-AS1* silencing enhances angiogenic sprouting in ECFCs. (**a**) Representative images of sprouting spheroids (scale bar = 100 µm). Quantification of sprout number (**b**) and cumulative sprout length (**c**) revealed increased angiogenic activity following *KLRK1-AS1* knockdown. Data are presented as mean ± SD; statistical significance was assessed using the Wilcoxon signed-rank test. *n* = 6 ECFC donors.

Angiogenic capacity was assessed using a spheroid sprouting assay. *KLRK1-AS1* silencing markedly enhanced sprouting activity compared with control conditions (Figure 5a).

Quantitative analysis revealed a 62% increase in the number of sprouts (*p* = 0.031; Figure 5b). Cumulative sprout length was also elevated, although this difference did not reach statistical significance (*p* = 0.094; Figure 5c). These findings indicate that loss of *KLRK1-AS1* promotes a pro-angiogenic ECFC phenotype.

## 4. Discussion

Our study identified lncRNA *KLRK1-AS1* as a regulator of ECFC function. Silencing *KLRK1-AS1* induced marked morphological alterations in ECFCs, accompanied by impaired cell–cell contacts and a pronounced reduction in monolayer barrier integrity. Strikingly, *KLRK1-AS1* depletion also enhanced angiogenic activity, reflected by an increased number of sprouts in the spheroid assay.

ECFCs are circulating endothelial progenitor cells capable of differentiating into mature endothelial cells. In vivo, they contribute to vascular repair and regeneration by homing to sites of endothelial injury [35] and participate in vascular growth and angiogenesis [36]. We previously demonstrated that *KLRK1-AS1* promotes wound healing in ECFC monolayers [23]. Building on this observation, the present study expands our understanding of *KLRK1-AS1* by examining its influence on additional key endothelial functions, namely proliferation, angiogenesis, and barrier integrity, to delineate its broader role in determining endothelial cell phenotype and function.

Indeed, we found that beyond impairing wound healing after monolayer injury, *KLRK1-AS1* silencing reduces monolayer resistance and induces a phenotypic shift toward an activated endothelial state, characterised by elongated cell morphology and enhanced angiogenic capacity. An elongated endothelial cell shape is typically associated with directed and accelerated migration, whereas rounded cells exhibit a more isotropic, random walk behaviour [37]. This is consistent with the observation that activation of endothelial cells by vascular endothelial growth factor (VEGF), a potent pro-angiogenic stimulus, promotes cell elongation [38]. The combination of reduced barrier integrity, elongated morphology, and increased sprouting strongly resembles the phenotype of activated endothelial cells. During angiogenesis, activated endothelial cells adopt a migratory state, loosen intercellular junctions [39,40] and elongate [38], while quiescent endothelial cells reinforce barrier function and establish stable cell–cell contacts [41]. Taken together, our findings suggest that *KLRK1-AS1* supports the maintenance of a quiescent, stable endothelial phenotype, whereas loss of *KLRK1-AS1* shifts ECFCs toward an activated, pro-angiogenic state. This highlights a potential role of *KLRK1-AS1* in preserving endothelial function and vascular homeostasis.

Impedance modelling demonstrated that *KLRK1-AS1* knockdown predominantly impaired cell–cell contacts, while cell–matrix adhesion remained unaffected. Endothelial barrier integrity relies heavily on cell–cell junctions, specifically tight and adherens junctions, formed between neighbouring endothelial cells, whereas cell–matrix contacts are primarily mediated by focal adhesions. Although both junctional systems contribute to overall barrier stability, inflammatory stimuli preferentially disrupt cell–cell junctions, leading to increased paracellular leakage [41]. This suggests that examining the role of *KLRK1-AS1* under inflammatory or pro-inflammatory conditions may provide valuable insights into its function in endothelial resilience and dysfunction.

Thus, endothelial barrier integrity and angiogenic activity represent two tightly interconnected yet functionally distinct aspects of endothelial biology. While intact intercellular junctions and low permeability are hallmarks of a quiescent, homeostatic endothelium, angiogenic activation requires transient loosening of cell–cell contacts, increased motility, and structural remodelling. Hence, the reduction in barrier function, the increase in angiogenic sprouting and the change in cell shape reflect a shift in endothelial activation in absence of *KLRK1-AS1*.

Non-coding RNAs have long been recognised as pivotal epigenetic regulators of cellular function in physiology and pathology, including endothelial function [42,43,44,45]. Among them, miRNAs that modulate mRNA or protein expression of endothelial cell-specific genes have been collectively termed angiomiRs [44,45,46]. A growing number of lncRNAs have also been implicated in the regulation of endothelial behaviour. Prominent examples include *MALAT1*, *H19*, Aerrie, *LASSIE*, and *MEG8*, which have been shown to modulate angiogenic capacity, migration, barrier integrity, proliferation, cell survival, and senescence of endothelial cells [47,48,49,50,51,52]. Thus, some lncRNAs promote endothelial function, some lncRNAs promote endothelial dysfunction, and some lncRNAs can act in both ways [22]. Although no unifying terminology exists for lncRNAs that regulate endothelial function, their relevance in vascular biology is increasingly recognised. By analogy to angiomiRs, which describe miRNAs involved in the regulation of angiogenesis and other endothelial cell functions, we propose that the term “angiolncRNAs” (angiolncs) may be useful as a descriptive concept to classify lncRNAs that influence endothelial behaviour. In this conceptual framework, *KLRK1-AS1* can be considered an angiolnc, as it contributes to the maintenance of endothelial quiescence and barrier stability while restraining excessive angiogenic activation.

*KLRK1-AS1* is an extremely versatile molecule, and the mechanisms through which it exerts its effects can currently only be speculated upon. Among other reported regulatory mechanisms, some *KLRK1-AS1* isoforms encode the peptide TP53LC04, which is induced by TP53 and suppresses cell proliferation [32]. We cannot exclude the possibility that TP53LC04 is also produced in ECFCs and becomes downregulated upon *KLRK1-AS1* silencing, potentially leading to enhanced cell proliferation. However, quantification of BrdU incorporation, a direct measure of DNA synthesis, as well as a monolayer capacitance, indirectly indicating proliferation, revealed no differences in proliferation between *KLRK1-AS1*-silenced cells and controls, suggesting that TP53LC04 is unlikely to modulate proliferation in our cell model; however, dedicated follow-up studies would be required to draw a definitive conclusion.

One limitation of our study is that, despite our finding that *KLRK1-AS1* influences endothelial barrier function, we did not investigate the underlying structural mechanisms such as cell junctions or intercellular contacts in detail. Therefore, it remains unclear whether the observed changes in barrier function are mediated by alterations in junctional proteins, cytoskeletal organisation, or other mechanisms regulating endothelial permeability. A further limitation is that we investigated the function of *KLRK1-AS1* exclusively through in vitro silencing, which prevents us from drawing direct conclusions about its in vivo relevance. Consequently, we can only speculate about the physiological or pathological consequences of reduced *KLRK1-AS1* expression in living organisms. Nevertheless, our previous finding that *KLRK1-AS1* is downregulated in ECFCs exposed to the pro-inflammatory environment of pregnancies with high gestational weight gain suggests a potential link between inflammatory stimuli and reduced *KLRK1-AS1* expression in humans [23]. This observation raises the hypothesis that *KLRK1-AS1* downregulation may promote an activated endothelial phenotype as a shared mechanism triggered by inflammation or other CVRF. To further explore this possibility, future studies should examine *KLRK1-AS1* expression and function in ECFCs derived from individuals with acute or chronic inflammatory conditions or endothelial dysfunction. Such investigations could provide deeper insight into the role of *KLRK1-AS1* in endothelial activation and its potential relevance for cardiovascular health.

## 5. Conclusions

Overall, our study demonstrates that *KLRK1-AS1* is involved in regulating endothelial function and can be classified within the growing group of angiolncs. Reduced *KLRK1-AS1* expression shifts ECFCs toward an activated, pro-angiogenic phenotype characterised by impaired barrier integrity and enhanced sprouting activity. These findings broaden current understanding of lncRNA involvement in endothelial physiology and pathology and CVD [53,54], and may, in the long term, help inform the development of novel therapeutic strategies targeting endothelial dysfunction [42,55,56].

## Data Availability

The original data presented in the study are openly available in Zenodo at https://doi.org/10.5281/zenodo.18030033.

## Supplementary Materials

The following supporting information can be downloaded at: https://www.mdpi.com/article/10.3390/life16020279/s1, Figure S1: Genomic map of *KLRK1-AS1*; Figure S2: Immunocytochemical stainings confirming ECFC identity; Figure S3: Reference images for the blinded observer rating.

## Abbreviations

The following abbreviations are used in this manuscript:

BrdU	5′-bromo-2′-deoxyuridine
CVD	Cardiovascular disease
CVRF	Cardiovascular risk factor
ECFC	Endothelial colony-forming cell
ECIS	Electrical cell–substrate impedance sensing
EPC	Endothelial progenitor cell
FBS	Fetal Bovine Serum
KLRK1	Killer cell lectin like receptor K1
KLRK1-AS1	Killer cell lectin like receptor K1-antisense 1
LMM	Linear mixed-effects model
lncRNA	Long non-coding RNA
miRNA	MicroRNA
OD	Optical density
SD	Standard deviation
siRNA	Small interfering RNA
VEGF	Vascular endothelial growth factor
VWF	Von Willebrand Factor
