# Supplementary material for: Downregulation of the Long Non-Coding RNA KLRK1-AS1 Disturbs Endothelial Barrier Integrity and Promotes Angiogenic Sprouting
Source: Life (Basel). 2026 Feb 5;16(2):279. doi: 10.3390/life16020279 (PMC12941382; doi:10.3390/life16020279)
Supplement: Supplementary file 1 [file life-16-00279-s001.zip › Supplementary Figure S2_r.pdf]

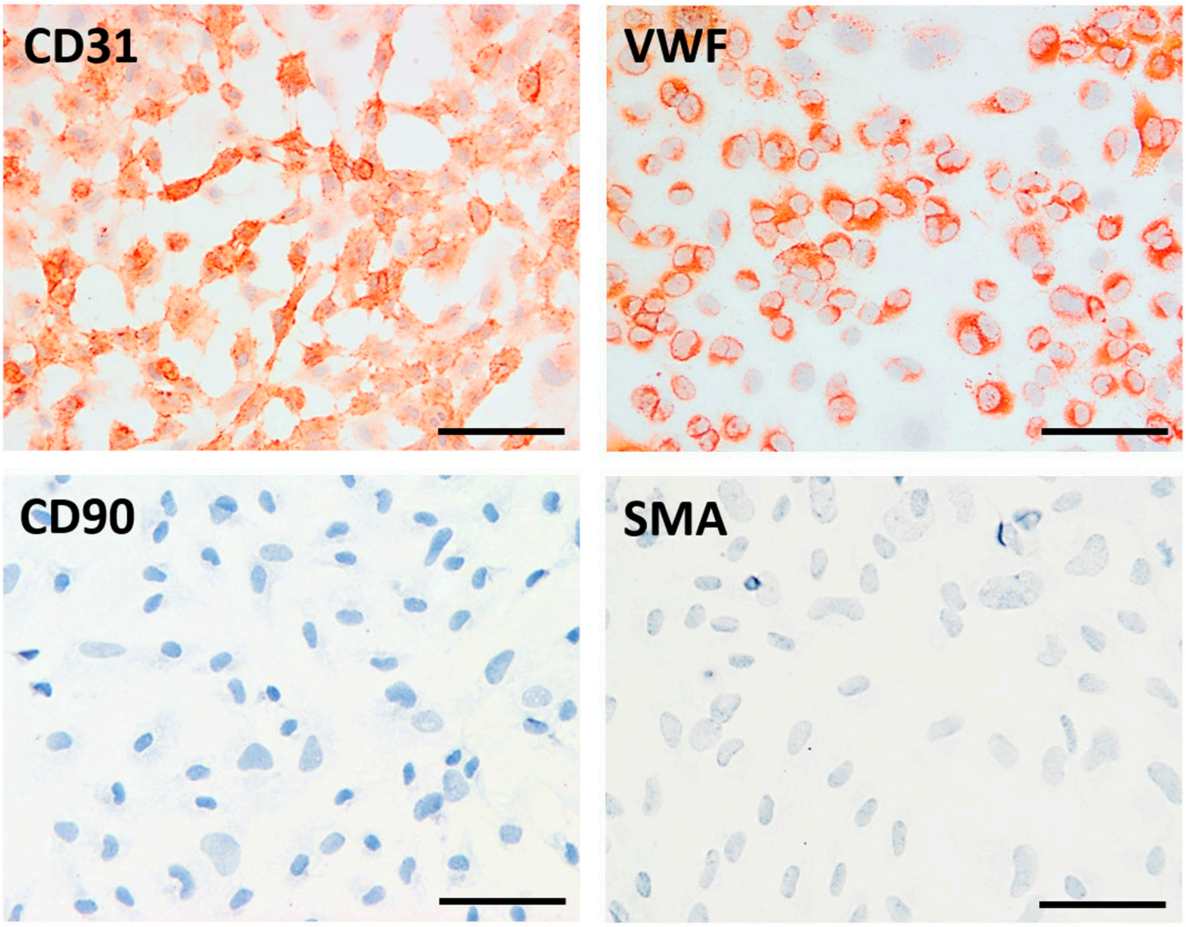

Supplementary Figure S2. Immunocytochemistry was routinely performed to confirm endothelial cell identity and purity, which was verified by positive staining for the endothelial cell markers CD31 (clone EN4, Monosan, Uden, the Netherlands) and von Willebrand factor (VWF; polyclonal, Agilent, Santa Clara, CA), together with the complete absence of staining for the fibroblast marker CD90 (AS02, Dianova, Hamburg, Germany) and the smooth muscle cell marker smooth muscle actin (SMA; clone M0851, Agilent). Immunocytochemical stainings were performed using the UltraVision LP Large Volume Detection System HRP (horseradish peroxidase) Polymer Kit (Thermo Scientific, Rockford, IL) according to the manufacturer's instructions. Scale bar = 100µm.
