# Supplementary material for: Downregulation of the Long Non-Coding RNA KLRK1-AS1 Disturbs Endothelial Barrier Integrity and Promotes Angiogenic Sprouting
Source: Life (Basel). 2026 Feb 5;16(2):279. doi: 10.3390/life16020279 (PMC12941382; doi:10.3390/life16020279)
Supplement: Supplementary file 1 [file life-16-00279-s001.zip › Supplementary Figure S3_r.pdf]

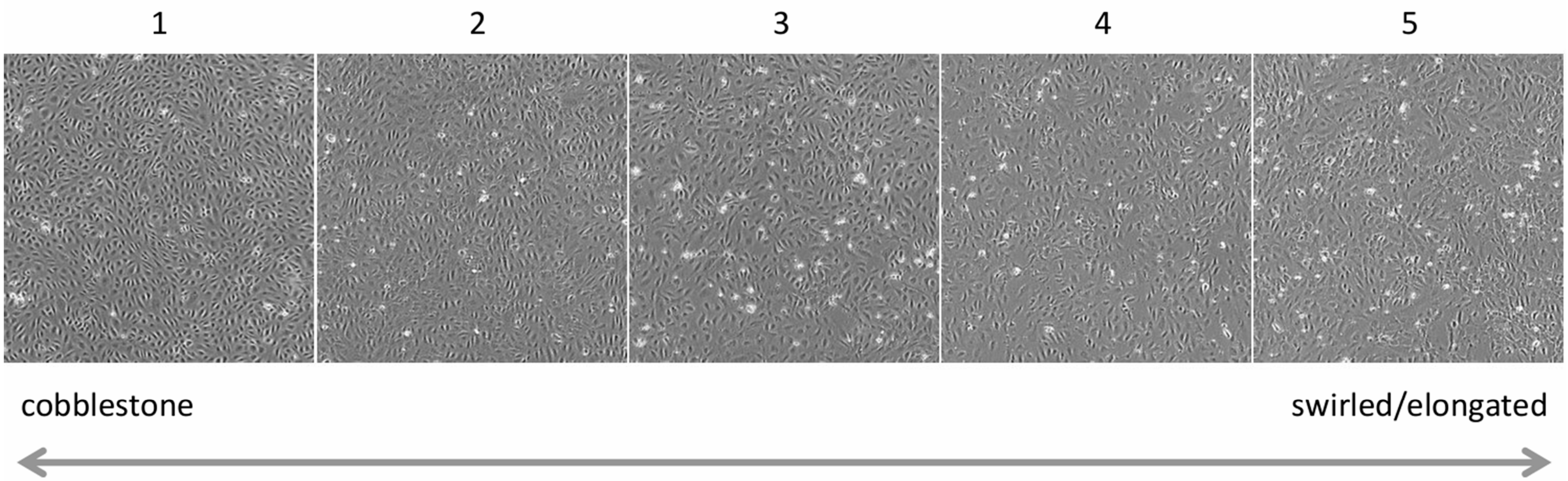

Supplementary Figure S3. Reference images used to guide grading of ECFC morphology in the blinded observer analysis.
