# Supplementary material for: Downregulation of the Long Non-Coding RNA KLRK1-AS1 Disturbs Endothelial Barrier Integrity and Promotes Angiogenic Sprouting
Source: Life (Basel). 2026 Feb 5;16(2):279. doi: 10.3390/life16020279 (PMC12941382; doi:10.3390/life16020279)
Supplement: Supplementary file 1 [file Supplementary Figure S1_r.pdf › Supplementary Figure S1_r.pdf]

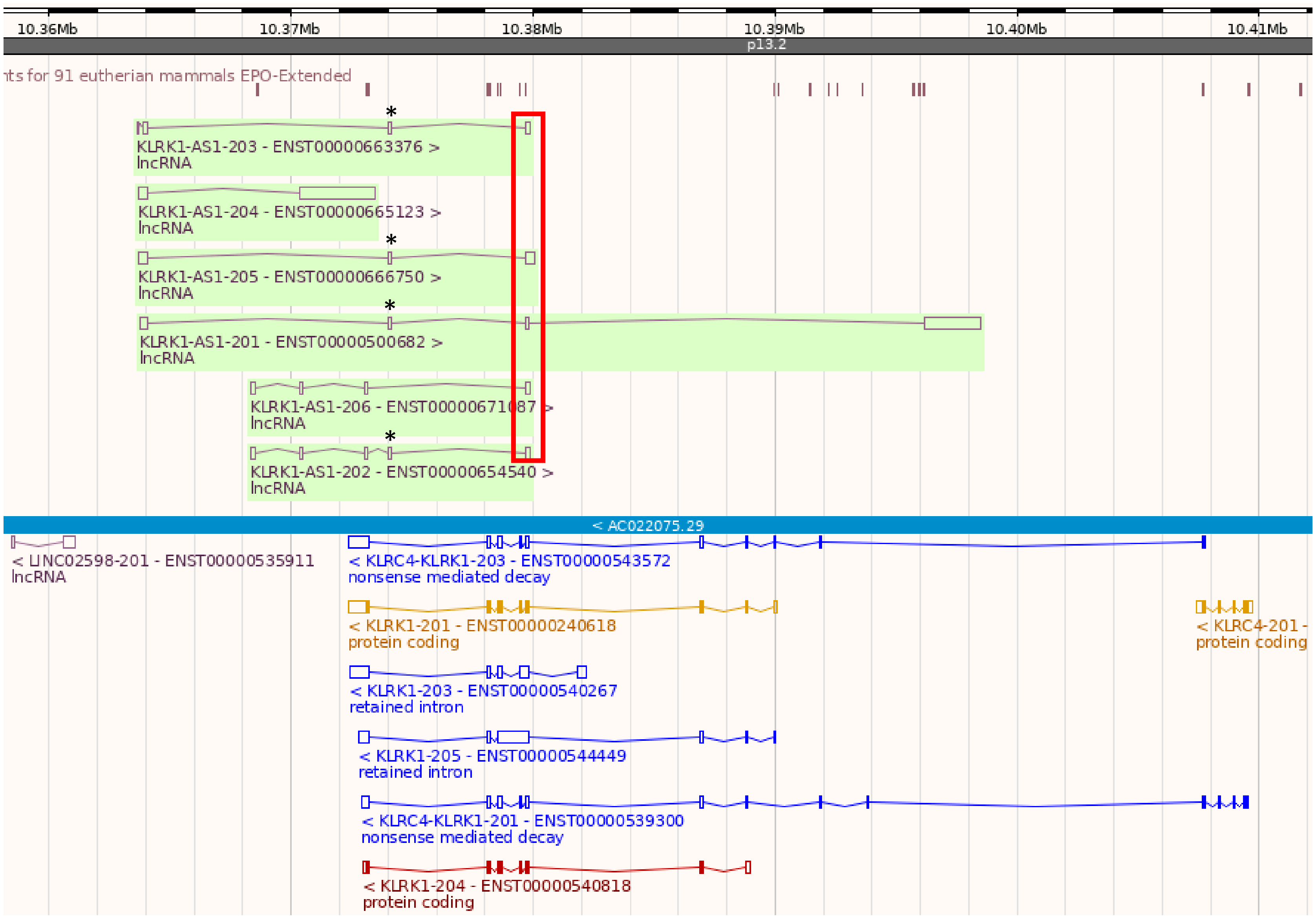

Supplementary Figure S1. *KLRK1-AS*1 genomic map. The *KLRK1-AS*1 isoforms are shown in green in the upper part of the figure. Isoforms of the host gene *KLRK1* are shown in the lower part of the figure. The *KLRK1-AS1* exons targeted by the specific siRNA used in the study are framed in red. The introns encoding the TP53-induced peptide TP53LC04 on *KLRK1-AS1* are marked with asterisks. The genomic region map was created on www.ensembl.org for *KLRK1-AS1* with the identifier ENSG00000245648.
